# Polyploid Giant Cancer Cells Are Frequently Found in the Urine of Prostate Cancer Patients

**DOI:** 10.3390/cancers15133366

**Published:** 2023-06-27

**Authors:** Laura Nalleli Garrido Castillo, Julien Anract, Nicolas Barry Delongchamps, Olivier Huillard, Fatima BenMohamed, Alessandra Decina, Thierry Lebret, Roger Dachez, Patrizia Paterlini-Bréchot

**Affiliations:** 1 Rarecells Diagnostics, F-75015 Paris, France; 2 National Institute for Health and Medical Research (INSERM), Institut Necker Enfants Malades-INEM, Université Paris Cité, F-75015 Paris, France; 3 Service d’Urologie, AP-HP, Hôpital Cochin, F-75014 Paris, France; 4 Service de Cancérologie, AP-HP, Hôpital Cochin, F-75014 Paris, France; 5 Service d’Onco-Urologie, Hôpital Foch, F-92150 Suresnes, France; 6 Innodiag, Pathology Laboratory, F-92100 Boulogne-Billancourt, France

**Keywords:** giant cells, polyploid giant cancer cells, PGCC, prostate cancer, urine, liquid biopsy, biomarkers

## Abstract

**Simple Summary:**

Recently, cells of large size called PGCC (Polyploid Giant Cancer Cells) have emerged as a pillar in cancer development and progression, possibly being the “first cells” from which the cancer starts. PGCC have been studied in cancer tissues from patients and in laboratory models. They have also been found in the blood, occasionally. By applying a method able to detect rare cells in urine, we found these PGCC in the urine of patients with prostate cancer. No study has ever published this finding. Our work is preliminary but deserves to be shared with the scientific community as it opens the way for more studies targeting the role of these PGCC and their possible use as an early and non-invasive marker of prostate cancer development.

**Abstract:**

Prostate cancer is the third cause of cancer-related deaths in men. Its early and reliable diagnosis is still a public health issue, generating many useless prostate biopsies. Prostate cancer cells detected in urine could be the target of a powerful test but they are considered too rare. By using an approach targeting rare cells, we have analyzed urine from 45 patients with prostate cancer and 43 healthy subjects under 50 y.o. We observed a relevant number of giant cells in patients with cancer. Giant cells, named Polyploid Giant Cancer Cells (PGCC), are thought to be involved in tumorigenesis and treatment resistance. We thus performed immune-morphological studies with cancer-related markers such as α-methylacyl-CoA racemase (AMACR), prostate-specific membrane antigen (PSMA), and telomerase reverse transcriptase (TERT) to understand if the giant cells we found are PGCC or other urinary cells. We found PGCC in the urine of 22 patients, including those with early-stage prostate cancer, and one healthy subject. Although these results are preliminary, they provide, for the first time, clinical evidence that prostate cancers release PGCC into the urine. They are expected to stimulate further studies aimed at understanding the role of urinary PGCC and their possible use as a diagnostic tool and therapeutic target.

## 1. Introduction

Prostate cancer is a commonly diagnosed urologic malignancy and the third cause of cancer-related death among men over 65 y.o. [1]. Despite the urgent need for new methods to detect prostate cancer early and non-invasively, cells from prostate cancer have been rarely identified by urine cytology. In fact, reports using either cytospin or ThinPrep^®^ urinary cytology have shown very few cases with prostate cancer cells in urine [2] and urine examination for prostate cancer is not recommended because of its low sensitivity. However, urine cytology could potentially allow early and non-invasive identification of prostate cancer, which is imperative, particularly in asymptomatic men, to plan for further analyses and follow-ups by urologists.

Polyploid Giant Cancer Cells (PGCC) are interesting cells which have been suggested to be the first cells in tumorigenesis [3]. It has been reported that environmental stresses may trigger an increase in cell size which switches proliferative mitosis to intranuclear replication. This process may restructure the cell genome to neoplastic transformation via the formation of PGCC. Thus, a change in cell size together with ploidy number switch may represent the most fundamental mechanism of how, on the one hand, somatic cells cope with environmental stresses for survival and, on the other hand, tumor development starts.

The field of PGCC is new and these cells have only been partially characterized. PGCC are generally rare cells in tumor tissues and are morphologically heterogeneous. In the blood, these cells may have a size ranging from 25 µm to 300 µm [4,5]. They may have one atypical nucleus or be multinucleated cells. In vitro models of giant cells (GC) showed that they can be three-to-ten times larger than regular cancer cells [6].

Several names have been used in the literature referring to them, including cancer-associated macrophage-like cells (CAMLs) [4], neoplastic-immune hybrid cells [7], Polyploid Giant Cancer Cells (PGCC) [6,8], polyaneuploid cells (PACCs) [9], blastomere-like cancer cells [10], osteoclast-like cancer cells [11], circulating giant tumor–macrophages fusion cells [12], hybrid cells [13], and pleomorphic cancer cells. It is unclear if different names correspond to different cell characteristics or not.

Interestingly, CAMLs have been shown to contain tumor molecules possibly derived from phagocytosis [7]. A high number of CAMLs detected before treatment was correlated with shorter overall and progression-free survival in pancreatic ductal adenocarcinoma (PDAC) patients [14]. In the blood, their presence has been related to poor prognosis, as well as to cancer progression in patients with prostate, breast, ovarian, and pancreatic cancer, among others [4,15]. However, PGCC have never been looked for in the urine of cancer patients.

We report the results of our study to detect GC non-invasively in patients with prostate cancer. By using ISET^®^-based cytology, we have found a high proportion of patients with prostate cancer having cancer-like GC in the urine compared to control subjects. Cancer GC have been described in giant cell carcinoma of the bladder, urothelial carcinomas, and transitional carcinoma of the bladder [16,17]. However, this was not the diagnosis in our patients. We did not find any reports about PGCC in urine associated with prostate cancer.

Therefore, we have studied these GC by morphological and immuno-morphological analyses to explore their possible cancer origin. We found that the majority of the GC express cancer markers.

Although we report preliminary data here, our study suggests that PGCC can be detected in the urine of a relevant proportion of patients with prostate cancer. Our results encourage further studies targeting PGCC in the urine as a possible biomarker of prostate cancer development.

## 2. Patients and Methods

In 2021, we started a research program aimed at developing a reliable urine-based test for the early detection of prostate cancer. In this setting, we have planned to study urine from patients with prostate cancer and from healthy subjects. During this study, we noticed that large multinucleated and mononucleated cells were frequently found in patients with prostate cancer.

We thus comparatively assessed at a single point in time the presence and features of GC detected in single urine samples from patients with clinically significant prostate cancer and from healthy subjects.

We studied samples from 45 patients with clinically significant prostate cancer followed at the Urology and Oncology departments of Cochin Hospital, Paris, France, and at the Urology department of Foch Hospital, Suresnes, France. Informed consent was obtained from all subjects before they provided a urine sample (Institutional Review Board-approved protocol, code 2020-A02712-37). No clinical follow-up was planned. All patients had been diagnosed with prostate adenocarcinoma by biopsy before or well, after urine collection (see below). The majority of patients had a Gleason score of 7 or higher (44/45 = 97.8%) and 13 out of 45 (28.9%) patients had metastasis. Metastases were diagnosed by imaging (bone scan). The average PSA serum level was 39.0 ng/mL. See Table 1 for clinical characteristics.

The inclusion criteria for prostate cancer patients were patients with newly diagnosed and untreated prostate cancer, not having been diagnosed with any other type of tumor before the inclusion; patients with metastatic prostate cancer on any imaging; and patients with high serum PSA level and/or a high risk of prostate cancer having French Social Security affiliation and agreeing to participate in this study.

Forty-three healthy subjects without known prostatic pathology such as benign prostatic hyperplasia (BPH) or prostatitis, under 50 y.o., and agreeing to participate in this study were also included. Healthy subjects had an average age of 32.4 y.o., ranging from 20 to 49. Informed consent was obtained from all subjects.

### 2.1. Urine Sample Collection and Stabilization

Single voided urine samples were obtained from patients with clinically significant prostate cancer prior to any treatment and without any short-term previous prostate stimulation such as digital rectal examination, prostatic massage, or prostate biopsy. There were 20 samples collected before prostate biopsy, while 25 samples were collected 2–4 weeks after biopsy (Supplementary Table S1). No urine samples presented macroscopic hematuria. Voided urine samples, including the first urine catch, were collected at any time of the day, directly into a suitable container, and fixed with modified Saccomanno’s fixative within the first 30 min after collection. Fixed urine samples were stored for up to 7 days. No predetermined urine volume was required for this exploratory study. The samples’ volume varied from 10 mL to 200 mL. After fixation, urine samples were conserved at room temperature and treated in the following 24 h or conserved at 4 °C for up to 7 days before treatment.

### 2.2. Human Cell Lines

PC-3 (prostate; PSA positive), VCaP (prostate; PSMA and PSA positive), LNCaP (prostate; PSMA, AMACR, and TERT positive), DU-145 (prostate; EpCAM positive), A375 (melanoma; vimentin and epithelial cocktail positive), HeLa (cervix; TERT and weak CD163 positive), and Daudi (B lymphoblast; negative control for all tested markers) cell lines were selected as positive and negative controls for the different ICC protocols (optimization and routine) (Table 2). The cell lines were purchased from ATCC^®^ (Manassas, VA, USA). Those cell lines were cultured in a complete medium containing DMEM with 10% FBS and 10 U/mL penicillin and streptomycin (Life Technologies, Waltham, MA, USA) in a 5% CO_2_ atmosphere at 37 °C. Cells were passaged every 3–5 days according to the growing speed. After cell detachment with trypsin, the cells were counted using Trypan Blue, added to urine samples collected from healthy volunteers, then fixed with modified Saccomanno’s fixative for at least 30 min and finally treated by ISET^®^ (Rarecells Diagnostics, Paris, France); or added to DPBS and fixed with 1.8% formaldehyde for 2 min at room temperature and processed alone.

### 2.3. Urine Processing

Fixed urine samples (volumes ranging from 10 mL to 200 mL) were processed through a proprietary method using a suitable ISET^®^ device and disposable cartridges (Rarecells Diagnostics, Paris, France). Briefly, urine was directly loaded into the cartridge without previous centrifugation nor sedimentation and treated at −10 kPa. After processing, ISET^®^ membranes were dried and stored at −20 °C until use.

### 2.4. Cells Analysis—Giemsa Stain and Immunocytochemistry (ICC)

A specific protocol was applied in order to analyze cell morphology and ICC labeling on the same cell. ISET^®^ membranes containing fixed cells from urine or human cell lines spiked in urine from healthy subjects were stained with Giemsa’s Azur eosin methylene blue solution (Merck 1.09204, Darmstadt, Germany), then digitized using the VS200 Slide Scanner (Olympus-LifeScience, Hamburg, Germany). Digital analysis (including cell count and cell size measurements) was performed using Olympus Image Analysis software version 2.1 (Hamburg, Germany).

Subsequently, cells were immunolabeled by ICC and then ISET^®^ membranes were digitized again. Comparative side-by-side image analyses of the same cells with cytological staining and with immunolabeling were carried out using Olympus Image Analysis software.

ICC protocols after Giemsa staining were applied to better-characterized GC. To this aim, ISET^®^ membranes were submitted to dual color ICC using EnVision™ G|2 Double stain System, Rabbit/Mouse (DAB+/Permanent Red, Agilent, Dako, Santa Clara, CA, USA).

Briefly, cells were hydrated with DPBS for 1 min. Double and single ICC protocols were started with 10 min of heat-induced antigen retrieval at 70 °C. Cell permeabilization was performed with Triton 0.1% in DPBS. Endogenous peroxidases were inactivated for 10 min. Primary antibodies (Table 2) were incubated for 1 or 2 h according to each protocol. After that, the polymer/HRP reagent was incubated for 15–30 min. DAB+ Working Solution was added for detection. After three washes with distilled water, spots were dried at room temperature. For the double-stain protocol, ISET^®^ membranes were immediately washed with washing buffer solution, the double-stain block solution was added and incubated for 10 min, second antibodies were incubated for 1 or 2 h, the Rabbit/Mouse (LINK) solution for 10 min, followed by 30 min with the polymer/AP reagent. Finally, the permanent red working solution was applied for signal detection.

Side-by-side images of the same cell after Giemsa staining and ICC labeling were submitted to cytopathological analysis.

Aside from the GC, we did not count all the cells on the spots, nor did we normalize the analyses based on urine cellularity or urine volume.

## 3. Results

### 3.1. Morphological Characterization of Polyploid Giant Cancer Cells in Urine Samples

To investigate the presence of PGCC in urine samples, we analyzed the cytomorphological characteristics of urinary cells enriched from 45 men diagnosed with prostate cancer and 43 healthy men 49 y.o. or younger.

PGCC were defined as cells with size equal or larger than 50 µm (maximum axis) and with cancer-like morphological characteristics: mononucleated or multinucleated cells (>10 nuclei) with nuclear hyperchromatism, high nucleo-cytoplasmic ratio, atypical nucleoli (number and size), and atypical cell shape and/or anisonucleosis.

**Figure 1:**
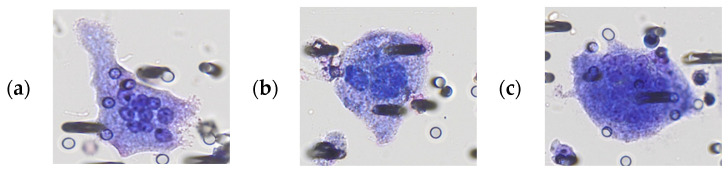

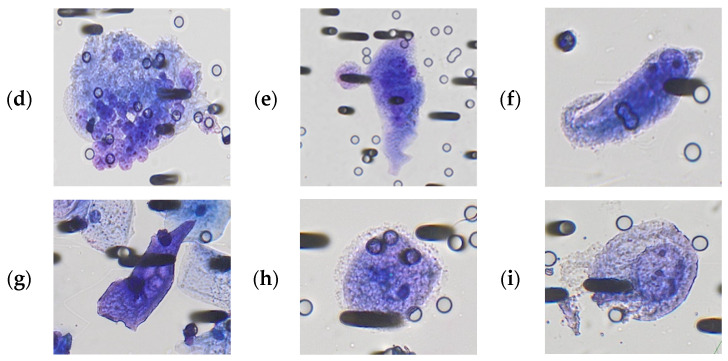
Giant cells from prostate cancer patients and healthy donors after Giemsa staining. (**a**–**f**) are representative images of the different PGCC morphologies observed in prostate cancer patients. Cell shapes: (**a**,**e**,**f**) tadpole; (**b**) round shape; (**c**) globular; (**d**) amorphous. (**g**–**i**) are representative images of cells observed in healthy donors’ urine samples.

Morphological analysis of urine samples from patients with prostate cancer revealed variable GC phenotypes including globular, oblong, spindle-like, round, tadpole, and amorphous shapes (Figure 1a–f). In healthy men, GC phenotypes included amorphous, round, globular, and oblong shapes (Figure 1g–i). Multinucleated cells with 10 or more nuclei were observed exclusively in patients with prostate cancer (6 out of 25 patients, 24%) and were considered to be GC with malignant features. GC from healthy subjects showed the presence of a regular and thickened outer contour of cytoplasm and similarities to urothelial cells.

**Figure 2:**
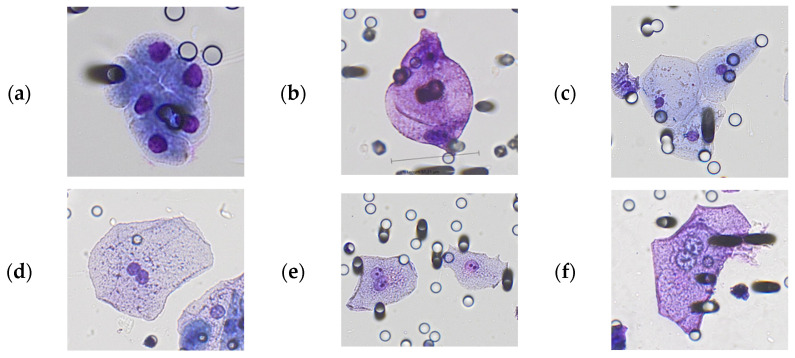
Representative images of normal cells present in urine samples. Cells stained with Giemsa’s Azur eosin methylene blue solution. Top (**a**–**c**): normal epithelial cells, bottom (**d**–**f**): umbrella cells.

Despite their frequency and size of 50 microns or larger (Figure 2), we did not count normal squamous epithelial cells (mainly from the distal urethra), urothelial cells (transitional epithelium from renal pelvis, ureters, bladder, and proximal part of the urethra), or cells with cytomorphological characteristics of umbrella cells (superficial layer covering basal and intermediate urothelial cells in the bladder) as GCs. Transitional cells from the intermediate and basal layer are characterized by round nuclei, smooth nuclear membrane, and high N/C ratio due to the small amount of cytoplasm (Figure 2a). Squamous epithelial cells are the largest cells in the urine sediment and are often considered as “contaminants” in urine cytology. They are flat cells with a single small, condensed nucleus, an opaque and uniform cytoplasm, and well-defined borders (Figure 2c). Umbrella cells are derived from the most superficial cell layer in the bladder urothelium. They are characterized by a finely vacuolated, transparent, textured cytoplasm (compared to squamous cells) and double or multiple nuclei with at least one prominent small nucleolus (Figure 2d–f). Umbrella cells can be multinucleated but, in general, with no more than five nuclei. Highly multinucleated cells may be observed in instrumented urinary specimens (e.g., bladder washing or brushing).

We did not observe a correlation between the quantity and/or type/characteristics of urinary cells and urine volume.

The morphological analysis allowed us to identify 64 GC with malignant features; thus, PGCC in 25 out of 45 patients (55.6%) with prostate cancer. In those patients, we also observed 64 GC with uncertain malignant features. In addition, we observed, in a further 7 patients, 47 GC exclusively with uncertain malignant features (without PGCC by morphology). On the other hand, we observed 23 GC with suspected malignant features (10 GC from 6 subjects) and with uncertain malignant features (13 GC from 5 subjects) in a total of 11 out of 43 healthy men (see Table 3).

### 3.2. Immunocytological Characterization of Polyploid Giant Cancer Cells in Urine Samples

**Figure 3:**
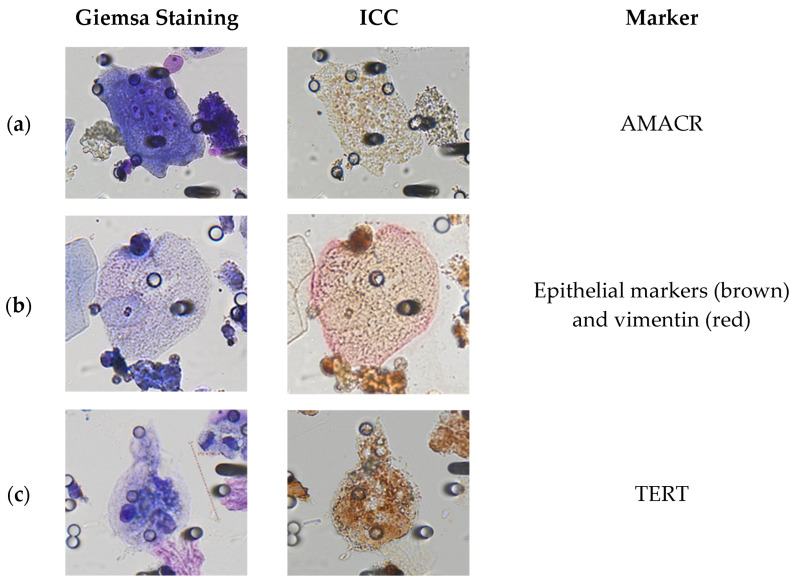

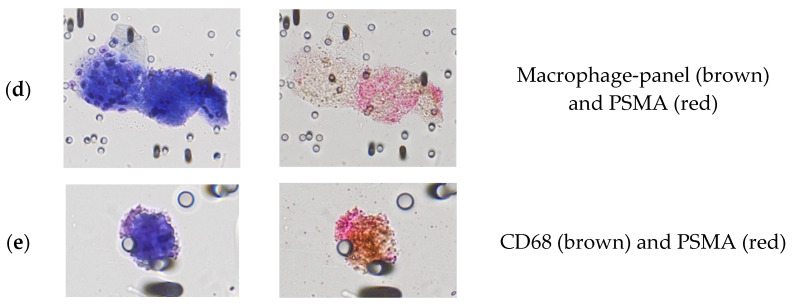
Characterization of PGCC from prostate cancer patients by Giemsa staining and ICC. Left panels correspond to Giemsa staining, while middle panels show the same cells after ICC. (**a**) multinucleated PGCC expressing AMACR; (**b**) mononucleated PGCC in EMT, positive to epithelial (brown) and vimentin (pink) markers; (**c**) multinucleated PGCC strongly positive to TERT (nuclear and cytoplasmic positivity); (**d**) multinucleated PGCC strongly expressing PSMA (pink) marker; (**e**) mononucleated PGCC expressing the macrophage marker CD68 (brown) with strong PSMA expression (pink). Visualization system: DAB (brown) and permanent-red (pink).

As reported in the Methods section, we performed an individual cell, side-by-side analysis of cytological and immunocytological aspects (Figure 3) to help the identification of PGCC. In fact, immunostaining often masks the cytomorphological aspects which can be the key to identifying tumor-like features. We observed that GC with malignant features often express macrophage-panel markers (CD68, CD163, CD11b) or epithelial markers (AE1/AE3, KL-1, EpCAM, E-cadherin) in combination with a second marker. PGCC were found to express cancer-related markers such as α-methylacyl-CoA racemase (AMACR) (Figure 3a), or telomerase reverse transcriptase (TERT) (Figure 3c). Other PGCC were found in epithelial–to-mesenchymal transition (EMT), as they expressed both epithelial markers (+) and vimentin (+) (Figure 3b). Finally, some multinucleated PGCC were positive to prostate-specific membrane antigen (PSMA), thus confirming their tumor nature and prostatic origin (Figure 3d). We observed PGCC co-expressing macrophage (CD68 or macrophage-panel) markers and PSMA (Figure 3e), possibly derived by the fusion of a macrophage cell with a prostate tumor cell.

The cytopathological criteria required to identify tumor cells are known to be strict. Therefore, when they are not clear-cut, or are only partially present, the cells are defined as having uncertain malignant features. In gynecological cytology, cells with uncertain malignant features are defined as ASCUS (Atypical Squamous Cell of Unknown Significance) because it is difficult to classify them as tumor or non-tumor cells. However, a large study has demonstrated that, for ASCUS cells’ correct classification, the combination of cell morphology with immunolabeling (double labeling p16-Ki67) is more accurate than either cell morphology or immunolabeling alone [18]. In the setting of cells with uncertain malignant features, the use of complementary techniques such as ICC can help the cells’ classification. In this study, we applied the same approach of combination of cytopathological criteria and immunolabeling characterization to confirm or not the tumor feature observed by cytopathology and to classify GC with uncertain malignant features.

By using the combination of tumor cytomorphology and ICC showing the presence of tumor-like markers, either EMT markers (epithelial+/vim+), PSMA, AMACR, or TERT, we found 50 PGCC from 22 prostate cancer patients, with an average number of 2.3 (range from 1 to 10) (see Table 3 and Table S1).

Besides PGCC, we also observed, in prostate cancers patients, the presence of smaller cells (<50 µm) expressing PSMA, AMACR, or other tumor markers, in variable amounts. However, the analysis of non-giant cancer cells in the urine of prostate cancer patients will be the subject of a following larger study and is not included in the present work.

Interestingly, we found the presence of large (≥50 µm) macrophage-like cells in the urine of prostate cancer patients.

The proportion of prostate cancer patients with PGCC was similar when urine was collected before (45%, 9 out of 20) versus after (52%, 13 out of 25) biopsy, showing that the PGCC we found after biopsy were not spread by the procedure. In fact, the urine sample was collected 2–4 weeks after biopsy (see Supplementary Table S1).

The ICC analysis of GC from heathy subjects using EMT markers, PSMA, and AMACR scored negative in all cases except in 1 healthy subject, who was found with 32 AMACR+ GC cells, thus classified as PGCC. We do not know if these PGCC are derived from a prostate cancer since they did not express prostate markers (PSA or PSMA) (see Supplementary Table S2).

In two healthy subjects, we also found 5 GC with EMT markers but normal cytomorphological features. Furthermore, no cell in those samples had malignant or uncertain malignant features. Thus, we did not consider the 5 GC as PGCC because EMT can also be a marker of cell renewal (see Section 4).

## 4. Discussion

Our study shows that giant cells (GC) having the characteristics of Polyploid Giant Cancer Cells (PGCC) are found in the urine of patients with prostate cancer. PGCC are rare cells that have been identified mostly in tumor tissues and studied by in vitro analyses. Some GC (called CAMLs) have also been found in the blood. Despite their big size, it is not trivial to distinguish them, in human samples, from non-cancer GC, mainly macrophages or inflammatory GC. However, extensive studies in cancer patients and in vitro have identified some GC as cancer cells able to give rise to tumors and tumor recurrence.

Our team works in the field of liquid biopsy, in particular, in the isolation and characterization of circulating tumor cells and circulating tumor GC from blood. In this setting, we have extended our studies to the research of prostate cancer-derived tumor cells in the urine of patients with prostate cancer. As a first observation, we found the presence of GC more frequently in the urine of prostate cancer patients than in control subjects. We thus conducted a detailed immune-morphological study of these GC to identify or exclude their tumor nature.

Giant polynucleated cells are very rare in urine; they can be superficial urothelial cells, including umbrella cells, macrophages, or cancer GC. Umbrella cells (also known as facet cells or superficial cells) are highly differentiated bi- or multinucleated cells, with a size ranging from 25 μm to 250 μm in diameter [19] located in the bladder urothelium. The total number of epithelial cells in urine (squamous and transitional cells) varies among men [20]. Giant multinucleated umbrella cells (with up to 10 nuclei) can be seen in urine but this finding is definitely rare. By the way, atypical umbrella cells can be found in the urine of patients with urothelial cancer and be of diagnostic help [21,22,23]; however, they have never been described in the urine of patients with prostate cancer and, in any case, umbrella-like cells in urine have never been associated with prostate cancer.

We defined as PGCC those cells with size equal or larger than 50 µm and having cancer-like cytomorphological aspects (such as anisonucleosis, nuclear hyperchromatism, high nucleo-cytoplasmic ratio, atypical nucleoli, and atypical cell shape), also expressing tumor markers (TERT, PSMA, AMACR, vimentin and epithelial markers, etc.) detected by immunolabeling.

The side-by-side cytomorphological and immunolabeling study helped us to identify the distinctive features of PGCC. In fact, during this study, some GC with uncertain malignant features according to morphological analyses, were shown to express tumor markers such as TERT, AMACR, or PSMA. As an example, the cell shown in Figure 3a could be mistakenly considered an atypical umbrella cell, but its expression of the AMACR marker showed its highly probable malignant nature. We have previously demonstrated the high specificity and limited sensitivity of cytomorphology in the field of circulating tumor cells. By studying the single cell morphological and genetic characteristics of CTC from patients with VHL-positive clear cell renal carcinoma, we found that cytomorphology had 100% specificity but much lower sensitivity [24]. In that study, we observed that all the cells with malignant features carried the VHL mutation in single cells’ analyses, thus were genetically proven tumor cells. However, also a relevant proportion of cells with uncertain malignant features were found to carry the same VHL mutation that was found blindly in the tumor tissue, showing that the presence of incomplete malignant features does not exclude the tumor cell nature. The same seems possibly true for some PGCC that we detected in urine. In the future, advanced molecular studies will be able to provide further diagnostic elements.

The EMT phenotype, which consists in the expression of epithelial markers and vimentin (epithelial+/vim+), is considered a tumor hallmark. However, it can also be related to embryonic development, skin wound healing, tissue regeneration, and renal fibrosis [25,26]. For example, EMT is required for re-establishing the skin barrier after a wound. Moreover, it has been shown in the context of bladder cell renewal after surgery. In healthy subjects, we found 5 GC with EMT markers but normal cytomorphological features. No cell in those samples had malignant or uncertain malignant features. Thus, we did not consider those cells as PGCC, but as cells with features associated with cell renewal.

We found one healthy subject with 32 AMACR + GC cells, which we thus classified as PGCC. Since we could not detect the expression of prostate markers (PSA or PSMA) in these PGCC, we do not know if they derive from prostate cancer or from another urogenital malignancy. We are now trying to obtain information on the genetic and clinical background of this subject in order to understand the finding of so many PGCC in his urine.

It is important to note that in this work, we did not include a control group made of matched-age men because in men older than 60 y.o., the probability of having undiagnosed prostate cancer is high [1]. Further studies are needed in order to include this population.

Interestingly, we found some PGCC (Figure 1d) similar to the already-described PGCC generating daughter tumor cells by budding. This characteristic of PGCC has been reported in vitro in primary cultures of ovarian cancer [6] and in cell lines derived from leukemic cells [27]. It is thought to be involved in the origin of PGCC-derived proliferating tumor cells in patients with cancer recurrence [9,28,29]. Although it is difficult to definitively demonstrate PGCC budding by images of cells detected in biological liquids from patients, published studies clearly indicate this possibility [27,28,30]. To our knowledge, this is the first report showing PGCC “budding-like” images in urine samples; thus, further studies are needed to explore the finding and related hypothesis.

By applying ICC analyses, we have found, in certain GC, the expression of tumor markers or a combination of them, including epithelial markers (AE1/AE3, KL-1, EpCAM, E-cadherin), macrophages markers (CD11b, CD68, CD163), mesenchymal marker (vimentin), and prostate tumor marker (PSMA), as well as cancer-related markers (AMACR, and TERT) helping PGCC identification. TERT expression has been shown, in tissue analyses, to be absent in patients with benign prostatic hyperplasia (BPH) but present in patients with prostatic intraepithelial neoplasia and prostate cancer [31]. Moreover, TERT expression has been used to detect CTC in blood samples [32], as well as urothelial carcinoma in the urine of patients with bladder cancer [33]. We observed a strong cytoplasmic expression of TERT in the PGCC found in urine samples. Cytoplasmic expression of TERT was already observed in non-clear cell and clear cell hepatocellular carcinoma [34,35], cervical cancer [36], prostate cancer [37], and urothelial carcinoma [33], suggesting possible telomere-independent functions of TERT under conditions of environmental stress such as hypoxia and oxidative stress. As a matter of fact, PGCC are thought to be generated in response to stress conditions.

An interesting finding of this work is the presence of large (≥50 µm) macrophage-like cells in the urine of prostate cancer patients. Macrophages are an important component of all prostate tumors; their presence is associated with cancer progression and clinical outcomes depending on their phenotype (either cytotoxic-M1 (CD68) or protumorigenic-M2 (CD163)). It has been shown that patients with a high density of M2 macrophages within the prostate tumor mass have a worse prognosis [38], while M1 activation can cause frequent cytokine storms that over time might damage prostate cells and ultimately trigger tumor development [39].

We observed PGCC expressing PSMA and macrophage markers (CD68 or macrophages cocktail), strongly suggesting their tumor nature and their possible origin from cell–cell fusion.

Cell–cell fusion is a common phenomenon related to physiological processes such as fertilization and tissue repair in which the two lipid bilayer membranes from two cells merge into one. It is known that cancer cells can also fuse with other cells such as leukocytes, fibroblasts, and macrophages (heterotypic cell fusion), as well as with other cancer cells (homotypic fusion) [40,41]. Cell fusion originates bi- or multinucleated cells harboring particular cell attributes of both parent cells. As discussed by Sutton and collaborators, it has been suggested that cell fusion between immune cells and cancer cells is a possible mechanism related to cancer progression, playing a role in the metastatic cascade [7]. Giant tumor–macrophage fusion cells (≥50 µm) circulating in the blood have been described to be associated with shorter disease-free survival and overall survival (*p* < 0.05) in patients with lung cancer [12].

Concerning the origin of fusion cells in prostate cancer, previous works using murine prostate cancer models showed the positive spatial relationship between macrophages and PSMA expression [42,43], suggesting the possibility that, in the prostate cancer tissue, PGCC with both a macrophage and a prostate tumor cell component can be generated by cell fusion.

Our study shows for the first time that PGCC with macrophage and PSMA markers can be present in the urine of patients with prostate cancer. Further studies are needed to explore their origin and clinical impact.

As explained, this work aimed to investigate if cancer giant cells, also called PGCC, are present in the urine of patients with prostate cancer (Gleason score equal or higher than 7). With direct PGCC counting and without any normalization based on urine cellularity or urine volume (which will be implemented in future studies), we found the presence of PGCC in 22 out of 45 (50%) cancer patients and 1 out of 43 healthy subjects, showing that cancer giant cells are present and detectable in the urine of prostate cancer patients. 

However, our search is not yet optimized as urine samples were not normalized. Urine samples are more difficult to normalize than blood samples, for which the collection of identical volumes is enough. Urine volume and cell dilution depend on the quality and quantity of previous drinking and/or physio-pathological conditions; thus, urine samples’ normalization needs specific rules to be identified and implemented. We have analyzed variable urine volumes among subjects (from 10 mL to 200 mL), which is expected to have an impact on the possibility to detect PGCC. Thus, further improvements in the approach are needed, which could also increase the sensitivity of PGCC detection.

The current study did not include any follow-up; thus, we could not investigate if there is a correlation between the number of PGCC, and/or their markers’ positivity with biochemical recurrence, or survival, or development of metastasis. However, we have now planned a second study targeting any patients undergoing prostatic biopsies, including their follow-up. This will allow us to analyze patients without prostate cancer (negative biopsies), and patients with Gleason 6 and Gleason ≥ 7, further exploring the presence of PGCC in patients with prostate cancer at an early stage, and in matched patients without prostate cancer. The follow-up, including after prostatectomy, will allow us to analyze the possible correlation of prostate cancer cells/PGCC in the urine with patients’ clinical outcome.

Urinary PGCC could be an early marker of prostate cancer. In fact, we found a similar proportion of prostate cancer patients positive for PGCC when urine was collected before (45%) and after (52%) biopsy. This finding indicates that (1) the results obtained in urine samples collected 2–4 weeks after biopsy were not biased by the biopsy, which is known to be associated with prostate cancer cells spread; (2) detection of PGCC before the biopsy could possibly be used as a way to identify patients with prostate cancer and confirm the need for biopsy, especially in problematic cases with serum PSA level in the grey zone, from 4 ng/mL to 10 ng/mL. As mentioned before, we have now planned a second study including patients with Gleason 6 to further explore the possible use of PGCC as an early hallmark of prostate cancer.

At the tissue level, so far, PGCC have been described in prostate cancer tissues from patients with resistance to treatment, and metastasis. As a matter of fact, pleomorphic giant cell prostate carcinoma is a rare tumor characterized by enlarged and pleomorphic cells frequently associated with resistance to prior therapy [44]. Furthermore, prostate cancer patients with focal pleomorphic giant cells have shorter overall survival [45], suggesting the key role of PGCC in cancer progression. Further studies are now needed to establish if PGCC detected in the urine could be an early marker of cancer and if they may have prognostic significance.

## 5. Conclusions

In conclusion, our study, although preliminary, provides clinical evidence that prostate cancers release PGCC into the urine. Our results raise several questions but also open the way to further investigations to explore the origin, characteristics, and clinical significance of PGCC detected non-invasively through a simple urine analysis.

## Figures and Tables

**Table 1:** Clinical and pathological characteristics of 45 patients with prostate cancer.

Clinical Parameter	Number (%) or Median (Range)
Age * average (range)	73 (55–94)
Serum PSA (ng/mL)	
Average	39.0
Median	12.0
Range	3.34–275
Unknown	2 patients
Gleason score	
6	1 (2.2%)
7	18 (40.0%)
≥8	26 (57.8%)
Metastasis	13 (28.9%)

* years.

**Table 2:** Primary antibodies used for cell characterization.

Antibody	Type	Control Positive Cell Line	Staining Pattern	Supplier
PSMA (3E6)	Mouse monoclonal	VCaP, LNCaP	Membranous/cytoplasmic	Agilent (Dako, Santa Clara, United States), M3620
AMACR (13H4)	Rabbit monoclonal	LNCaP	Cytoplasmic granular	Agilent (Dako, Santa Clara, United States), M3616
Panel–Macrophage markers (CD11b, CD68, CD163)	Rabbit monoclonal	HeLa	Cytoplasmic	Abcam (Cambridge, UK), ab254013
Vimentin (SP20)	Rabbit monoclonal	A375	Cytoplasmic	ThermoFisher Scientific (Waltham, MA, USA), MA5-14564
Panel–Epithelial markers (AE1/AE3, KL-1, EpCAM, E-cadherin)	Mouse monoclonal	A375, DU-145	Cytoplasmic and membranous	Agilent, M3515 Santa Cruz Biotechnology (Dallas, TX, USA), SC-58825, SC-25308, SC-8425
TERT (2D8)	Mouse monoclonal	LNCaP, HeLa	Nuclear/cytoplasmic	Thermo Fisher, MA5-16033
PSA (EP1588Y)	Rabbit monoclonal	PC-3, VCaP	Cytoplasmic/ membranous	Invitrogen, (Waltham, MA, USA), MA5-14470

**Table 3:** Table showing the total number of cancer patients and healthy subjects with urinary giant cells and with PGCC identified by cytomorphology and ICC (N° of cells in parentheses).

	Number	With Urinary Giant Cells	With Urinary PGCC
Prostate cancer patients	45	32 (175)	22 (50)
Healthy donors	43	11 (23)	1 (1)

## Data Availability

The datasets generated during and/or analyzed during the current study are available from the corresponding authors on reasonable request.

## Supplementary Materials

The following supporting information can be downloaded at: https://www.mdpi.com/article/10.3390/cancers15133366/s1, Supplementary Table S1. Prostate cancer patients’ data; Supplementary Table S2. Healthy donors.
