# Supplementary material for: Polyploid Giant Cancer Cells Are Frequently Found in the Urine of Prostate Cancer Patients
Source: Cancers (Basel). 2023 Jun 27;15(13):3366. doi: 10.3390/cancers15133366 (PMC10340459; doi:10.3390/cancers15133366)
Supplement: Supplementary file 1 [file cancers-15-03366-s001.zip › cancers-2434089-SI.pdf]

Table S1. Prostate cancer patients' data.

Prostate Cancer Patient	Gleason Score	Urine Volume Collected	Collection (Before or After Biopsy)	Number of PGCC	Markers
1	4+3	110	After	1	Vimentin / PSMA
2	4+4	60	After		
3	4+5	140	After	4	CD163 / Epithelial markers / Vimentin
4	3+4	30	After		
5	5+4	75	After	1	CD68-PSMA
6	3+3	150	After	1	Epithelial markers/Vimentin
7	4+4	140	After		
8	4+3	95	After		
9	4+3	130	After	1	Epithelial markers / CD163
10	3+4	15	After	1	Macrophages markers/PSMA
11	4+4	30	After		
12	3+4	60	After		
13	3+4	60	After	1	AMACR
14	4+4	50	After	10	CD68/PSMA / TERT
15	4+3	80	After	3	Macrophages markers/PSMA
16	4+5	150	After	3	EpCAM /CD163
17	4+5	65	After		
18	4+5	115	After		
19	4+4	70	After	3	TERT
20	4+5	75	After		
21	5+4	80	After		
22	4+4	80	After	4	Epithelial markers/Vimentin
23	5+4	75	After		
24	4+4	120	After	2	AMACR
25	3+4	45	After		
26	4+5	200	Before		
27	4+5	30	Before	1	PSA/PSMA
28	4+4	30	Before	1	PSA/PSMA
29	4+3	20	Before		
30	4+4	30	Before		
31	4+3	80	Before		
32	4+3	10	Before	4	Macrophage markers/PSMA
33	4+3	25	Before		
34	4+5	55	Before		
35	4+4	25	Before	2	Macrophage markers/Epithelial markers
36	4+4	20	Before	1	Macrophage markers/Epithelial markers
37	4+3	20	Before		
38	4+3	30	Before	2	Macrophage markers / Epithelial markers
39	4+3	100	Before		
40	4+3	40	Before	1	Epithelial markers/Vimentin
41	4+4	100	Before		
42	4+4	40	Before		
43	4+3	40	Before	1	Macrophage markers/PSMA
44	4+5	60	Before		
45	4+4	30	Before	2	Macrophage markers/PSMA

Table S2. Healthy donors .

ID	Urine Volume	Number of PGCC	Marker
1	80	32	AMACR
2	160	Zero	
3	15	Zero	
4	30	Zero	
5	70	Zero	
6	40	Zero	
7	120	Zero	
8	140	Zero	
9	125	Zero	
10	180	Zero	
11	85	Zero	
12	90	Zero	
13	80	Zero	
14	170	Zero	
15	130	Zero	
16	80	Zero	
17	30	Zero	
18	65	Zero	
19	75	Zero	
20	110	Zero	
21	70	Zero	
22	35	Zero	
23	65	Zero	
24	75	Zero	
25	70	Zero	
26	80	Zero	
27	80	Zero	
28	120	Zero	
29	150	Zero	
30	25	Zero	
31	120	Zero	
32	110	Zero	
33	60	Zero	
34	65	Zero	
35	60	Zero	
36	75	Zero	
37	200	Zero	
38	25	Zero	
39	125	Zero	
40	150	Zero	
41	120	Zero	
42	125	Zero	
43	100	Zero	
